# The Treatment of Tuberculous Laryngitis

**Published:** 1907-10-26

**Authors:** 


### Laryngology and Rhinology.

### THE TREATMENT OF TUBERCULOUS LARYNGITIS.

The treatment of laryngeal tuberculosis is fraught with especial difficulty, for it is nearly always merely a complication of an advanced stage of pulmonary phthisis, and neither thorough asepsis nor complete immobility of the organ is obtainable.

### Old Lines of Treatment.

Until some twenty years ago laryngeal tuberculosis was universally considered to be quite hopelessly incurable, and the beginning of a rapidly approaching end. When Krause and Hering, in 1885 and 1887, introduced local treatment by lactic acid and curettage, and demonstrated that complete healing of the larynx could be obtained, their results were hailed with great enthusiasm; this doubtless overstepped the mark at times, and led to the routine treatment with caustics and curette of many hopelessly advanced cases. The result was to bring these methods into disrepute, and all such local treatment was condemned by many laryngologists, especially in England. As generally happens in such cases, it was finally seen that the proper course of treatment lies between the two extremes, and it is now universally recognised that, while the routine local treatment of advanced consumptives is both useless and cruel, healing can be obtained in a very fair proportion of properly selected cases.

Tuberculosis attacks the larynges of patients in an early stage of pulmonary phthisis more often than is generally realised, and is common in chronic consumptives, who, but for this, have many years of life and work in prospect; in such cases the laryngeal affection can often be much improved and sometimes healed by active treatment.

### General Treatment.

In addition to local measures, general treatment is of the greatest value, and cases do best under open-air treatment at a sanatorium; even advanced tuberculous laryngitis is no contra-indication to open-air methods. Rest for the voice is of the greatest value, especially when the interior of the larynx is affected, and loud whispering is as great a strain as speech; improvement often occurs if complete silence be maintained, but it should be remembered that this is a severe and depressing measure, only to be employed where there is a fair chance of cure. It is important to allay cough as far as possible, for, unless this be done, the larynx cannot be kept at rest. Fog and damp do not seem to affect the larynx injuriously, but of all things a dusty and windy climate is the most harmful.

General treatment, however, should be assisted by local measures; it is rare that even early laryngeal disease becomes healed by general treatment alone, and the proportion of cures is undoubtedly increased when local treatment is used as well; on the other hand, it is not very uncommon to obtain healing by local therapy in the out-patient department of a hospital.

### Points in Local Treatment.

For superficial lesions the application of lactic acid maintains the first place. The strength of the solution and the frequency of the application must vary with the degree of reaction; it is often well to use it daily and in concentrations of about 50 per cent. It must not be painted on, but firmly rubbed in. The addition of carbolic acid 10 per cent., as advised by Lake, makes the application less painful.

Other escharotics are in use, but with all the secret of success is to use them strong enough, often enough, and hard enough; they are tolerated far better than might be expected.

### Curetting.

Hering's curettes are useful, especially for granulations and ulcers in the inter-arytenoid space, either at the beginning of treatment, or later if they do not improve with pigments; in using the curette one should attempt to remove as much as possible of the disease with one stroke; considerable force is necessary, and, after guiding the instrument into position with the mirror one may apply the left hand to the shank to act as a fulcrum.

### Injections.

But all such methods are useless in the treatment of massive infiltration beneath intact epithelium, such as occurs on the arytenoids and epiglottis. The injection of escharotics with a syringe has been employed, but is not free from the danger of necrosis, and galvano-cautery puncture has not proved very efficacious in this region. The removal of the parts with punch-forceps gives far more satisfactory results, and the wounds heal readily; though as much as possible should be taken away, it is not necessary to excise the infiltration as completely as if it were a cancer, for the remaining swelling shrinks rapidly, and the disease is opened up to the action of lactic acid, which should be applied subsequently. In advanced cases, where no cure is possible, the use of punch-forceps has great value for the relief of dysphagia, and in such cases the application of lactic acid should be omitted. For such operations thorough cocainisation is necessary, but local anæsthesia is not needed before the application of pigments, and the frequent use of cocaine is to be avoided.

### The Galvano Cautery.

In certain cases of tuberculous laryngitis the galvano-cautery is useful, especially in infiltration about the ventricular bands and ventricle of the larynx, which is too deeply situated for successful treatment with pigments; a finely pointed cautery should be plunged deeply into the affected area, and several punctures made at each sitting.

*(To be continued.)*

